# Exploring veterinary students’ awareness and perception of zoonoses risks, infection control practices, and biosecurity measures in Ethiopia

**DOI:** 10.3389/fvets.2024.1385849

**Published:** 2024-07-09

**Authors:** Ndungu S. Nyokabi, Lisette Phelan, Johanna F Lindahl, Stefan Berg, Emmanuel Muunda, Adane Mihret, James L. N. Wood, Henrietta L. Moore

**Affiliations:** ^1^Institute for Global Prosperity, University College London, London, United Kingdom; ^2^School of Geography, University of Leeds, Leeds, United Kingdom; ^3^Environmental Economics and Natural Resources Group, Wageningen University and Research, Wageningen, Netherlands; ^4^International Livestock Research Institute (ILRI), Nairobi, Kenya; ^5^Department of Medical Biochemistry and Microbiology, Uppsala University, Uppsala, Sweden; ^6^Department of Clinical Sciences, Swedish University of Agricultural Sciences, Uppsala, Sweden; ^7^Bernhard Nocht Institute for Tropical Medicine, Hamburg, Germany; ^8^Armauer Hansen Research Institute (AHRI), Addis Ababa, Ethiopia; ^9^Department of Veterinary Medicine, University of Cambridge, Cambridge, United Kingdom

**Keywords:** veterinary training, one health, biosecurity measures, occupational risks, animal health

## Abstract

Universities and colleges are often regarded as playing a key role in educating veterinarians and animal health workers who advise farmers on herd health and animal husbandry. However, to date, studies examining veterinary students’ knowledge of zoonotic diseases of public health importance and the source of this knowledge, as well as their preparedness to respond to these diseases, have focused on the Global North rather than the Global South. This study takes Ethiopia as a case study in exploring veterinary medicine students’ knowledge of zoonosis risks, infection control practices and biosecurity measures, recognizing that it is imperative to reconcile national-level veterinary education curricula with emerging global trends, such as One Health-focused training. This training advocates for a collaborative, interdisciplinary response at local, national, and international levels to the adverse impact of zoonotic diseases on animal health and productivity, and human and environmental health. Data for this study were collected through a pre-tested online questionnaire administered to 154 veterinary students from several universities in Ethiopia. The findings of this study suggest veterinary students were aware of the public health risks posed by zoonoses and the important role that collaboration between the disciplines of human and animal health can play in addressing zoonoses and emerging health risks. However, students demonstrated poor knowledge of the need to adopt infection control measures (ICPs) and biosecurity measures to reduce occupational risks and prevent within and between herd transmission of infection. Moreover, students’ vaccination rates against zoonotic diseases associated with occupational risks, such as rabies, were low. The results of this study suggest that there are currently gaps in Ethiopia’s veterinary curriculum and that enhancing veterinary students’ access to information regarding infection control practices and biosecurity measures could contribute to reducing their future occupational exposure to zoonoses. This study highlights the policy implications of the current veterinary medicine curriculum in Ethiopia and the scope for aligning the curriculum with important global initiatives, such as One Health-focused training.

## Introduction

Veterinarians contribute to global public health in the areas of prevention and control of animal diseases, emerging infectious diseases, zoonotic and non-zoonotic disease surveillance, food safety and security, environmental health, global health security, and basic and applied medical research (1). Veterinarians are engaged in various roles including herd health, animal husbandry procedures, disease screening and surveillance, vaccination, treatments and surgical procedures, providing advice to a range of stakeholders, and animal welfare management (2).

Universities and veterinary colleges educate veterinarians and animal health workers (1) to provide a solid clinical knowledge base and relevant diagnostic and surgical skills to solve a broad spectrum of health problems around the world, including but not limited to those involving animals (1, 3). Veterinary training equips practitioners with relevant skills to support and share responsibility for the protection of human health and well-being in diverse fields including food safety, environment, biodiversity, and bioterrorism preparedness (1). Historically, veterinarians have contributed to the prevention and control of epidemics such as bovine spongiform encephalopathy and foot-and-mouth disease in the United Kingdom, West Nile virus in the United States (1), and Rift Valley fever (RVF) in East Africa (4). These epidemics impacted human and animal health, the economy and societal well-being and were finally contained through the valuable contribution of veterinarians (1, 4).

In their day-to-day occupational activities, veterinary students may be exposed through their professionally supervised training process to zoonoses such as brucellosis, bovine tuberculosis, rabies, Hendra virus and Q-fever because of their close contact with infected and dead animals through ingestion and inhalation, via conjunctiva or physical contact (5–8). It is, therefore, crucial to train veterinary students in disease prevention and control through the adoption of infection control practices (ICPs) (6). ICPs are also referred to as personal biosecurity measures (9). Biosecurity measures are widely used to refer to a set of measures that stop the spread of disease onto or out of an area where farm animals are present (10). Generally, ICPs are any methods employed to stop a disease or infection from spreading across environments, animals and people (11), including those in direct or indirect contact at the farm level, such as fellow staff, farmers or their family members (6, 8). Similarly, animal health practitioners can serve as agents of disease transmission or as a bridge for disease as they move from one farm to another attending to sick animals (12). Although in other parts of the world, there has been research on ICPs, there is a paucity of studies in sub-Saharan Africa investigating veterinary students’ knowledge, perceptions, and adoption of ICPs (13–16).

This study uses Ethiopia as a case study because veterinary training is growing in most emerging economies (16, 17). Training of animal health assistants in Ethiopia started in 1963 with a two-year diploma program and the first Faculty of Veterinary Medicine was founded in 1979 at Addis Ababa University (AAU) (17). There are now 10 universities accredited to offer veterinary medicine degree programs (18). As of 2006, Ethiopia had 616 veterinarians, 3,993 animal health assistants and animal health technicians, and 1,375 community-based animal health workers, with over 87% of the veterinarians and 98% of the animal health assistants and technicians working in the public sector which shows the need to train more skilled veterinarians given the vast size of Ethiopia landmass and large number of farmers they have to serve (16, 17, 19). Currently, the quality and access to animal health services is a major challenge for farmers in Ethiopia (19–22). There are just a handful of studies on the Ethiopian veterinary profession (16, 17, 19), and to the best of our knowledge, no recent study has investigated the training of veterinary medicine, the expected education outcomes, the exposure to zoonoses (zoonotic risks), adoption of ICPs and sources of information. This study, therefore, aimed to explore the knowledge of veterinary students in Ethiopia, regarding their zoonoses risks, ICPs and sources of information.

## Methodology

This national survey of veterinary students was conducted via an online questionnaire between April and June 2021 and covered veterinary medicine students across all 10 universities across Ethiopia. This study was objectively investigating the training of veterinary students and as such did not cover other people involved in the veterinary world, including practicing veterinarians and lecturers. The questionnaire was designed based on a literature review of students’ knowledge of occupational risks, knowledge of personal ICPs and sources of veterinary information (16, 19, 23–26). This study employed an online questionnaire to enable participants to answer questions at their convenient time. We relied on convenience and purpose sampling to reach as many students as we could due to the travel restrictions related to COVID-19 that were present at the time that this research was conducted.

The questionnaire was pre-tested with five veterinary students, and adjustments and corrections were made based on their feedback. The pre-tested questionnaire survey consisted of both open and closed questions with options for the students to add more information (see Supplementary material). The questions aimed at collecting demographic information such as place and year of study, the student’s knowledge of occupational risks, knowledge of personal ICPs, and sources of veterinary information. The questionnaire was hosted by Survey monkey® and took 30 min to complete. For enrolment, a questionnaire link was sent to students by email via student associations, class representatives and other veterinary organizations. Additionally, with help from practicing veterinarians and student organizations, the link was shared on social networking sites like Facebook, X (formerly Twitter) and WhatsApp that students used. The study purposively looked for experienced students at an advanced level of veterinary studies mainly third-year to sixth-year, and automatically excluded those in lower levels of study as they were not exposed to practical training with live animals, tissues and/or were not working in farm or clinical settings.

The survey was designed as an opt-in survey whereby taking part was considered as signed consent. No personal data was collected in this survey as an ethical consideration to avoid participant identification and/or victimization. The survey questionnaire was deleted from the hosting website after the data collection phase was completed. This study had ethics clearance from the University College London Research Ethics Committee (UCL-REC) approval number 19867/001 and the Armauer Hansen Research Institute (AHRI) and ALERT hospital AHRI/ALERT Ethics Review Committee (AAERC) approval Protocol number PO-(46/14).

### Data management and analyses

The data collected during the survey was downloaded as an Excel document. Statistical analyses mainly descriptive statistics were undertaken using R statistical software.

The research design, data collection, analysis and writing were done collaboratively by all the authors.

## Results

Table 1 presents the demographic characteristics of the participating veterinary students across the various veterinary schools at Ethiopian Universities. In total 154 veterinary students took part in this study. The majority of respondents were male students. The majority of respondents were in their 5^th^ year of training and already doing work placements.

**Table 1:** Characteristics of participant veterinary students in this study (*n* = 154).

		*n* (%)
Average age	Years (mean ± SD)	24.8 ± 1.3
Gender	Male	102 (66.2%)
	Female	52 (33.8%)
Home region	Oromia Region	57 (37.0%)
	Harari Region	23 (14.9%)
	Sidama Region	9 (5.8%)
	Addis Ababa (city)	11 (7.1%)
	Southern Nations, Nationalities and Peoples’ Region	12 (7.8%)
	Gambela Region	7 (4.6%)
	Somali Region	3 (2.0%)
	Dire Dawa (city)	6 (3.9%)
	Amhara Region	21 (13.6%)
	Afar Region	3 (2.0%)
	Benishangul-Gumuz Region	1 (0.7%)
	Tigray Region	1 (0.7%)
Year of study	3	22 (14.3%)
	4	26 (16.9%)
	5	79 (51.3%)
	6	27 (17.5%)
University	Addis Ababa University	21 (13.6%)
	Gambela University	1 (0.6%)
	Gonder University	34 (22.1%)
	Haremaya University	21 (13.6%)
	Hawassa University	7 (4.5%)
	Jijiga University	6 (3.9%)
	Jimma University	5 (3.2%)
	Mekelle University	3(1.9%)
	Samara University	5 (3.2%)
	Wolita Sodo University	25 (16.2%)
	Wollega University	26 (16.9%)

Table 2 presents the veterinary students’ perceptions of the risks of zoonoses, and the importance of collaboration across the animal-human health sectors to ensure good health in society. Students were aware of the risks of zoonoses and the important role of collaboration between human and animal health. The students were aware of the occupational risks that farmers, farm workers and veterinary professionals were exposed to when working and interacting with livestock. They were also aware of the food safety and disease risks faced by consumers of animal-source products. Finally, they thought that climate change could exacerbate the risk of zoonoses and drive the emergence of new zoonoses.

**Table 2:** Veterinary students’ perceptions of zoonoses (*n* = 154).

What level of importance do you attribute to the following statements?	Extremely important [*n* (%)]	Very important [*n* (%)]	Moderately important [*n* (%)]	Slightly important [*n* (%)]	Not at all important [*n* (%)]
How important is collaboration between human and animal healthcare providers for ensuring public health?	101 (65.6%)	32 (20.8%)	12 (7.8%)	8 (5.2%)	1 (0.6%)
How important is monitoring and detecting zoonoses or outbreaks in animal populations for preventing human infection?	104 (67.5%)	34 (22.1%)	12 (7.8%)	4 (2.6%)	-
How important is maintaining ecosystem and environmental health for protecting human and animal health?	96 (62.3%)	37 (24.0%)	13 (8.4%)	8 (5.2%)	-
How important is preventing human encroachment in preventing the emergence of new human and animal diseases?	93 (60.4%)	40 (26.0%)	17 (11.0%)	3 (1.9%)	1 (0.6%)
How important is addressing climate change for preventing the emergence of new human and animal diseases?	94 (61.0%)	41 (26.6%)	11 (7.1%)	6 (3.9%)	2 (1.3%)

To what extent do you agree or disagree with the following statements?	Strongly agree	Agree	Neither agree nor disagree	Disagree	Strongly disagree
Consumers of animal products are at risk of contracting zoonoses	43 (27.9%)	91 (59.1%)	8 (5.2%)	5 (3.2%)	7 (4.5%)
Animals can serve as disease sentinels for human health	83 (53.9%)	46 (29.9%)	12 (7.8%)	4 (2.6%)	9 (5.8%)
Climate change, directly and indirectly, impacts human and animal health	47 (30.5%)	85 (55.2%)	12 (7.8%)	3 (1.9%)	7 (4.5%)
Veterinary and animal health professionals are at risk of contracting zoonoses	42 (27.3%)	90 (58.4%)	11 (7.1%)	2 (1.3%)	9 (5.8%)
Farmers and farm-workers are exposed to the risk of contracting zoonoses	57 (37.0%)	74 (48.1%)	13 (8.4%)	2 (1.3%)	8 (5.2%)

### Knowledge perception and the use of infection control practices and biosecurity measures

When asked to define biosecurity which involved a choice of multiple answers for each respondent, 126 (81.8%) students replied it meant preventing the entry of pathogens or diseases onto a farm, 129 (83.8%) students thought it is managing diseases and/or pathogens within a farm, 120 (77.9%) students thought it was preventing the exit of diseases/pathogens from the farm, 103 (66.9%) students said it is the general security to prevent theft of animal, 85 (55.2%) students replied protecting workers from disease and only 4 (2.6%) students were unsure of the definition.

Table 3 presents a summary of veterinary students’ knowledge and adoption of ICPs and biosecurity measures for disease prevention. The students were aware of the risks associated with medical waste. They cleaned their hands after touching animal tissue, and fluids and properly disposed of veterinary wastes such as needles and razors. Although students were vaccinated, it was mainly the standard immunizations received in childhood. There was low vaccination uptake for diseases associated with occupational risks such as rabies. Moreover, there was low use of personal equipment (PPE) such as gloves, overcoats and surgery gowns. PPE was mostly cleaned with normal washing detergents without much disinfection.

**Table 3:** Infection control practices (ICPs) and biosecurity measures adopted by veterinary students for disease prevention (*n* = 154).

		*n* (%)
How do you clean and take care of your reusable material and equipment (needles, syringes, scalpels, razors)?	Cleaning with detergent only	94 (61.0%)
	Cleaning with detergent and soaking in disinfectant	35 (22.7%)
	Cleaning and autoclaving (hot sterilization)	25 (16.2%)
How often do you wash your hands while working with animals?	After each animal	64 (41.6%)
	After each lot	56 (36.4%)
	After each cattle farm	33 (21.4%)
	No regular cleaning	1 (0.6%)
How do you wash or clean your hands after working with animals?	Clean with water only	122 (79.2%)
	With a soap	146 (94.8%)
	With an antibacterial soap	113 (73.4%)
	With hand sanitizer	80 (51.9%)
How do you dry your hands?	With re-usable towel	38 (24.7%)
	With a paper towel	115 (74.7%)
	Other (please specify) air drying	1 (0.6%)
Do you carry a yellow container for medical waste when you visit farms (i.e., in your car or bag)		133 (86.4%)
How do you dispose of empty medicine packaging and vaccine flasks/vials?	Yellow container for medical waste in the lab	127 (82.5%)
	Domestic trash can	129 (83.8%)
	Collected by a specialized company	132 (85.7%)
	Glass waste container	132 (85.7%)
	Government waste place	67 (43.5%)
Have you been vaccinated for zoonotic and other likely occupational diseases?	Yes, I have been vaccinated	132 (85.7%)
	Yes, for Tuberculosis	4 (2.6%)
	Yes, for Tetanus	10 (6.5%)
	Yes, for Rabies	19 (12.3%)
How often do you change your overall, apron or overcoat?	I wear reusable clothing and change it as soon as it is visually dirty	32 (20.8%)
	I wear reusable clothing and change it after specific ‘dirty’ work	55 (35.7%)
	I wear reusable clothing and change it after every cattle farm	32 (20.8%)
	I wear reusable clothing and change it every day	29 (18.8%)
	I use a set of disposable clothing for each cattle farm	2 (1.3%)
	I use disposable clothing (i.e., different set per cattle herd/group)	1 (0.6%)
	I use clothing provided by the farmer	3 (1.9%)
When performing surgeries, what do you wear?	Disposable calving gowns	15 (9.7%)
	Washable calving gowns under ordinary circumstances, but disposable gowns in case of known septic risk	98 (63.6%)
	A washable gown used of calving (calving gowns)	41 (26.6%)
What do you use when washing your work clothes and linen?	Detergent	56 (36.4%)
	Disinfectant	104 (67.5%)
	Soap	147 (95.5%)
	Coldwater	122 (79.2%)
	Warm water	50 (32.5%)
	Hot water	11 (7.1%)

Table 4 presents a summary of the biosecurity measures adopted by veterinary students when visiting farms and attending livestock to minimize and prevent the risk of introduction and transmission of diseases within and between farms. The results of Table 4 show that students had a low usage of protective clothing and equipment (PPE) and the use of clean medical equipment to prevent disease transmission associated with touching animals, fluids and tissues while undertaking practical lessons, farm visits and internships. The students had a low adoption rate of important ICPs such as disinfection of work boots aimed at the prevention of farm-farm and between-herd transmission of livestock diseases. The use of the same needles and examination gloves within a farm poses a risk as it can lead to within-herd disease transmission.

**Table 4:** Biosecurity measures that veterinary students would adopt when working on farms (*n* = 154).

	*n* (%)	*n* (%)	*n* (%)	*n* (%)	*n* (%)	*n* (%)
How would you take care of your work boots?	After each farm	Before each farm	After and before each farm	Between two buildings on the same farm	Only when they are visually dirty	Never
Brushing	14 (9.1%)	12 (7.8%)	27 (17.5%)	81 (52.6%)	20 (13.0%)	0
Use of water jet (if provided at the farm)	3 (1.9%)	9 (5.8%)	35 (22.7%)	83 (53.9%)	18 (11.7%)	6 (3.9%)
Cleaning with soap	9 (5.8%)	5 (3.2%)	39 (25.3%)	55 (35.7%)	43 (27.9%)	3 (1.9%)
Disinfection	7 (4.5%)	9 (5.8%)	48 (31.2%)	25 (16.2%)	63 (40.9%)	2 (1.3%)
Stepping through foot bath or stepping on the foot mat (if provided at the farm)	2 (1.3%)	8 (5.2%)	34 (22.1%)	54 (35.1%)	47 (30.5%)	9 (5.8%)

How often do you replace the following disposable materials and equipment?	After each animal	After each herd	After each farm	Every day	Less frequently than every day
Needles for injections	26 (16.9%)	17 (11.0%)	21 (13.6%)	89 (57.8%)	1 (0.6%)
Sample collection needles	16 (10.4%)	15 (9.7%)	20 (13.0%)	103 (66.9%)	–
Syringes for injections	17 (11.0%)	11 (7.1%)	17 (11.0%)	102 (66.2%)	7 (4.5%)
Scalpel and razor blades	19 (12.3%)	9 (5.8%)	29 (18.8%)	83 (53.9%)	14 (9.1%)
Examination gloves	14 (9.1%)	13 (8.4%)	46 (29.9%)	59 (38.3%)	22 (14.3%)
Full-arm veterinary gloves	12 (7.8%)	13 (8.4%)	52 (33.8%)	48 (31.2%)	29 (18.8%)

Table 5 presents a summary of the adoption of ICPs and biosecurity measures that students are trained to take as veterinarians are meant to take, to minimize occupational exposure to zoonoses in the course of their work. Students had the perception that it was important to use PPE for personal protection from zoonoses.

**Table 5:** Infection control practices (ICPs) and biosecurity measures that veterinary students perform to minimize occupational exposure to disease when working on farms (*n* = 154).

In each of the following scenarios, please indicate the ICPs you would implement in examining animals and performing procedures.	Hand wash after contact	Gloves only	Boots/shoe disinfection	Overalls only	Gloves and overalls	Gloves, overalls, respiratory masks, and goggles	Not sure
	*n* (%)	*n* (%)	*n* (%)	*n* (%)	*n* (%)	*n* (%)	*n* (%)
Post-mortem examination of a cow	24 (15.6%)	7 (4.5%)	2 (1.3%)	5 (3.2%)	12 (7.8%)	103 (66.9%)	1 (0.6%)
Routine examination of a cow	13 (8.4%)	17 (11.0%)	2 (1.3%)	7 (4.5%)	12 (7.8%)	103 (66.9%)	–
Cow with acute watery diarrhoea	11 (7.1%)	11 (7.1%)	10 (6.5%)	7 (4.5%)	10 (6.5%)	103 (66.9%)	2 (1.3%)
Aborted fetal material from a cow	4 (2.6%)	11 (7.1%)	7 (4.5%)	11 (7.1%)	27 (17.5%)	91 (59.1%)	3 (1.9%)
Cow with dystocia (calving problems)	3 (1.9%)	11 (7.1%)	8 (5.2%)	15 (9.7%)	57 (37.0%)	46 (29.9%)	14 (9.1%)
An animal with a multidrug-resistant urinary tract infection (after a number of treatments with different drugs)	46 (29.9)	3 (2.0)	57 (37.0)	15 (9.7)	11 (7.1)	8 (5.2)	14 (9.1)
Entering a farm	48 (31.2)	5 (3.3)	47 (30.1)	16 (10.4)	4 (2.6)	13 (8.4)	21 (13.6)

Table 6 presents a summary of the main sources of information that veterinary students use. The majority of students relied on a range of sources to get information on zoonoses, ICPs, biosecurity measures, One-Health, antibiotic resistance, new emerging treatments, and animal disease outbreaks. There is a strong reliance on personal networks such as peers and colleagues. There is also a reliance on online sources to source information regarding new treatments, disease emergence and new emerging technologies.

**Table 6:** Main sources of information for veterinary students (*n* = 154).

	Education curriculum	Colleagues and friends	Mass media (TV, Radio, newspaper)	Scientific journals	Online searches (i.e., Google)	Social networks (i.e., Facebook, Twitter, Instagram, LinkedIn)	Government communication	Professional association and subscriptions
Zoonoses	24 (15.6%)	64 (41.6%)	96 (62.3%)	98 (63.6%)	105 (68.2%)	98 (63.6%)	49 (31.8%)	12 (7.8%)
Biosecurity measures and/or ICPs	27 (17.5%)	59 (38.3%)	86 (55.8%)	107 (69.5%)	104 (67.5%)	103 (66.9%)	53 (34.4%)	18 (11.7%)
One-Health	24 (15.6%)	54 (35.1%)	91 (59.1%)	100 (64.9%)	104 (67.5%)	95 (61.7%)	52 (33.8%)	20 (13.0%)
Antibiotic resistance	29 (18.8%)	57 (37.0%)	108 (70.1%)	106 (68.8%)	111 (72.1%)	92 (59.7%)	51 (33.1%)	18 (11.7%)
New treatments	20 (13.0%)	58 (37.7%)	99 (64.3%)	97 (63.0%)	104 (67.5%)	84 (54.5%)	42 (27.3%)	17 (11.0%)
Animal disease outbreaks	25 (16.2%)	64 (41.6%)	97 (63.0%)	98 (63.6%)	100 (64.9%)	100 (64.9%)	60 (39.0%)	13 (8.4%)

Table 7 presents a summary of the ease of accessing information on various topics and from different sources. Students perceived that it was not easy to access information on antibiotic resistance, new treatments, and animal disease outbreaks. Access to information from other sources was perceived as easy, particularly from training curriculum, peers (colleagues and friends), mass media (TV, Radio newspaper), and online searches such as Google and social media (Facebook, Twitter, Instagram, LinkedIn).

**Table 7:** The ease of accessing information sources (*n* = 154).

How easy is it to get information on the following topics?	Very easy	Easy	Moderately easy	Difficult
	*n* (%)	*n* (%)	*n* (%)	*n* (%)
Zoonoses	42 (27.3%)	55 (35.7%)	50 (32.5%)	7 (4.5%)
Personal ICPs	18 (11.7%)	74 (48.1%)	55 (35.7%)	7 (4.5%)
Farm biosecurity measures	20 (13.0%)	74 (48.1%)	49 (31.8%)	11 (7.1%)
One-Health	14 (9.1%)	71 (46.1%)	56 (36.4%)	13 (8.4%)
Antibiotic resistance	16 (10.4%)	57 (37.0%)	67 (43.5%)	14 (9.1%)
New treatments	20 (13.0%)	41 (26.6%)	76 (49.4%)	17 (11.0%)
Animal disease outbreaks	27 (17.5%)	32 (20.8%)	68 (44.2%)	27 (17.5%)

How easy is it to get information from the following sources?	**Very easy**	**Easy**	**Moderately easy**	**Difficult**
Training Curriculum	19 (12.3%)	61 (39.6%)	63 (40.9%)	11 (7.1%)
Colleagues and friends	20 (13.0%)	78 (50.6%)	49 (31.8%)	7 (4.5%)
Mass media (TV, Radio newspaper)	27 (17.5%)	62 (40.3%)	54 (35.1%)	11 (7.1%)
Scientific journals	27 (17.5%)	56 (36.4%)	63 (40.9%)	8 (5.2%)
Online searches, e.g., google	41 (26.6%)	50 (32.5%)	56 (36.4%)	7 (4.5%)
Social media (Facebook, Twitter, Instagram, LinkedIn)	37 (24.0%)	55 (35.7%)	55 (35.7%)	7 (4.5%)
Government communication	21 (13.6%)	64 (41.6%)	57 (37.0%)	12 (7.8%)
Professional association and subscriptions	15 (9.7%)	71 (46.1%)	55 (35.7%)	13 (8.4%)

## Discussion

This study explored students’ perception of zoonoses risks; understanding of ICPs and biosecurity measures; disposal of medical waste materials; uptake of vaccines; and access to information that could reduce the occupational risk associated with exposure to zoonotic diseases. Globally, discussions are ongoing among veterinary medicine educators and administrators regarding the need to update ‘traditional’ veterinary curricula to address public health challenges faced at local, national and international levels, from the emergence of new zoonotic diseases to the re-emergence of endemic zoonoses, i.e., Rift Valley fever, SARS, pandemic influenza H1N1 2009, Yellow fever, Avian Influenza (H5N1) and (H7N9), West Nile virus, and Middle East Respiratory Syndrome coronavirus (MERS-CoV) (27). These discussions reflect a growing recognition that farmers’ ability to realize desirable animal health and productivity outcomes, as well as human and environmental health outcomes, is contingent on their access to quality services provided by well-trained and skilled veterinary and animal health professionals (28). Veterinary students’ ability to grasp and recall knowledge, for example, regarding zoonotic disease risks and antibiotic stewardship; make rational decisions; and communicate effectively with farmers directly impacts their ability to transition from an educational setting to a public or private practice setting (29). As students’ knowledge and skills base are shaped, in part, by the veterinary education curricula followed, the results of this study underscore that there is a need to better align veterinary training with emerging global trends, such as One Health-focused training that fosters collaborative transdisciplinary and interdisciplinary thinking to realize animal, human, and environment health (30–32).

### Perceptions of zoonoses risk among veterinary students at Ethiopian universities

The results of this study suggest that only a minority of students would rely on ICPs to prevent exposure to occupational zoonoses and recommend their use by farmers (Tables 3, 6). Previous research has shown that students face occupational exposure to zoonotic diseases due to their handling of live animals and/or their tissue (15, 26, 33). Practicing students could potentially disseminate zoonotic pathogens to their relatives or the animals they are treating during placements and internships, and thus the use of ICPs and hygienic measures is important to prevent disease spread (34). Veterinarians typically have higher seroprevalence for zoonoses than the general population due to their occupational exposure (3, 34). The results of this study affirm that it is important to ensure proper training of students on occupational risks related to zoonoses exposure in day-to-day activities, as has previously been suggested in the literature (3, 6, 15).

### Veterinary students’ understanding of ICPs and biosecurity measures

The findings of this study suggest a low perception of the importance of biosecurity measures adoption among students. This is concerning from an occupational safety and public health perspective given that biosecurity-related behavior can prevent disease transmission (5–7). Jackson and Villarroel (2012) observed that most veterinarians suffer from a zoonotic disease early in their careers, or even during their time spent at veterinary school. Training can prepare veterinary students for occupational risks associated with exposure to zoonoses in their day-to-day lives and incentivize their adoption of ICPs which reduce and prevent zoonoses risks (6, 15, 26). Studies suggest that training on critical biosecurity strategies, such as sanitation and disinfection procedures, as well as visitor and workflow policies, are key to disease prevention and control strategies (3). The results of this study affirm that, in the context of zoonoses prevention, the importance of using PPE, being vaccinated, and implementing ICPs should be emphasized in the training of veterinary students (26). Veterinarian students’ attitudes towards personal protection as practicing veterinarians can be changed or improved and rendered sustainable over time by the veterinary curricula followed (6, 15, 28, 34). As veterinarians are role models for farmers, veterinary students’ behavior based on knowledge and skills derived in an educational setting also has implications for farmers’ adoption of ICPs and biosecurity measures (35, 36).

The findings of this study indicate a low perception among veterinary students of the importance of implementing biosecurity measures when visiting farms and attending to livestock. Students’ behavior may increase the risk of the transmission of zoonotic diseases within and between farms (3). To minimize and prevent such risks at farms, it is crucial to ensure that visitors, including veterinary students, adopt biosecurity measures and use PPE when visiting farms and attending livestock (3, 6, 15). Biosecurity measures, such as the use of PPE and disinfection of work boots, have been recommended for the control of within- and between-herd transmission of zoonoses (23, 37). Although it can be expensive to use different PPE equipment for each animal, there is a need to ensure medical equipment such as syringes, needles and gloves are disinfected properly after use for each animal to minimize the risk of disease transmission within and between herds (23, 37).

### Disposal of medical waste materials

The results of this study suggest that students were aware of the zoonoses transmission risks associated with handling medical waste. The findings also highlight the need to train students on the proper disposal of medical waste materials, such as needles, razor blades and animal tissue, to avoid environmental contamination (38). Environmental contamination can serve as a reservoir for pathogens and can expose humans and animals to zoonotic diseases.

### Veterinary students’ access to information regarding zoonoses

The findings of this study reveal that veterinary students acquired information from various sources beyond the training veterinary curriculum followed, including peers (colleagues and friends), mass media (TV, Radio newspaper), online search engines (Google) and social networks (Facebook, Twitter, Instagram, LinkedIn). These information sources enable veterinary students to easily access and triangulate information which facilitates their decision making. Increased access to the internet and mobile phones and the emergence of online learning platforms have enabled students to accumulate knowledge beyond that which is taught in a classroom setting. It is, however, concerning that veterinary students believed that it was not easy to access information on antimicrobial resistance (AMR), new treatments and animal disease outbreaks, given the importance of these topics in Ethiopia and beyond (16, 18, 23). AMR results in therapeutic failures and increases lengths and/or cycles of treatment which has negative downstream impacts on animal welfare, food security, and public health (39). Access to information on animal disease outbreaks is crucial to managing and containing outbreaks and safeguarding animal, human and environmental health and livelihoods (19, 20). Ensuring that veterinarians and veterinary students have access to information on new treatments available in the market is key to enabling them to make informed decisions on treatments and prescriptions (19, 25).

### Policy implications

This study highlights the imperative for veterinary medicine educators to include material on zoonotic disease risks in their veterinary science degree programs, particularly focusing on biosecurity measures (i.e., hygienic measures and the use of ICPs) as a way of developing a culture that prevents occupational exposure during training and, later, in practice. In line with global initiatives, there is a need for One Health-focused training that fosters collaborative efforts of multiple disciplines working locally, nationally and globally to improve health human, animal and environmental health (30). Given the impact of zoonoses on livestock and humans, there is a need to bridge human health and animal health education and training systems (40). Students in Ethiopia could benefit from being informed about and engaging with the principles of One Health in the context of their university and college-level education and subsequent professional training, as recommended by the American Veterinary Medical Association (30). Currently, the Africa One Health University Network (AFROHUN), the International Livestock Research Institute (ILRI), and the One Health Research, Education and Outreach Centre in Africa (OHRECA) project are working to transform the training environment and approaches in African universities and capacity building through collaborative curriculum design and peer-peer benchmarking (31, 32).

The education that veterinary students receive should ensure that they are well-prepared for the job market (28). The results of this study show that there is a need to ensure that veterinary students receive holistic training that encompasses (i) laboratory diagnostics skills; (ii) practical skills that enable them to identify and respond to AMR and treat livestock diseases; and (iii) clinical skills required to practice reproductive and preventive medicine. The emergence of antimicrobial resistance is driven by the overuse of antimicrobials in human and/or veterinary medicine (41). Implementing One Health training through interdisciplinary training in the form of practical workshops, in-practice training, and external training with practitioners from other related disciplines can ensure veterinary students are equipped to respond to emerging health challenges such as AMR and new emerging zoonoses (28, 30). One Health can enable collaboration and exchange of information among human, animal and environmental health practitioners and could facilitate sharing of scares resources such as laboratories and laboratory expertise (40, 42). Realizing One Health and interdisciplinary training in veterinary schools hinges on the allocation of adequate resources and support by relevant public and private veterinary sector stakeholders (17, 28).

### Study limitation

In this study, we did not have the exact number of students enrolled in Ethiopian universities. We relied on convenience and purpose sampling to reach as many students as we could taking into account the travel restrictions related to COVID-19 that were present at the time that this research was conducted. An online questionnaire enables participants to answer questions at a time that is convenient and takes as much time as needed to respond. Online questionnaires render participation research more accessible to individuals with the internet or electronic devices such as phones and/or computers. Although the main objective of this sampling approach was to draw a diverse sample from across the population of university and college students in Ethiopia, a self-selection bias led to the majority of respondents being from the major universities in large cities where students tended to have a strong online presence and were active on social networks.

## Conclusion

The findings of this study suggest that there are currently gaps that need to be bridged in the teaching of veterinary students in Ethiopia, particularly related to the use of ICPs and biosecurity measures and the extent to which the ‘traditional’ veterinary education curricula could and should be improved to address public health challenges faced at local, national and international levels, from the emergence of new zoonotic diseases to the re-emergence of endemic zoonoses. Curriculum standardization and capacity training in line with the One Health concept could contribute to enhancing veterinary students’ access to information regarding infection control practices and biosecurity measures could contribute to reducing their future occupational exposure to zoonoses. Increasing the availability of personal protection equipment, such as gloves, could also increase the likelihood that students are in a position to engage in practical training and gain knowledge and skills beyond the realm of the theoretical education received. This study makes an important contribution to the literature on veterinary students’ knowledge of zoonotic diseases of public health importance and the source of this knowledge, as well as their preparedness to respond to these diseases, in the Global South. The findings of this study suggest that universities and colleges play an important role in equipping students with the skills and knowledge that enable them to make informed decisions on treatments and prescriptions and prevent within and between herd transmission of zoonotic infection. Against a backdrop of the emergence of new zoonotic diseases and the re-emergence of endemic zoonoses, it can be expected universities and colleges will continue to constitute a critical link in the translation of veterinary knowledge into practice, with veterinary medicine educators through their delivery of an updated education curriculum ensuring that veterinary students are in a position, in their subsequent careers as veterinarians and animal health practitioners, to effectively address the public health risks posed by zoonoses and enhance animal, human, and environmental health.

## Data availability statement

The raw data supporting the conclusions of this article will be made available by the authors, without undue reservation.

## Author contributions

NN: Conceptualization, Data curation, Formal analysis, Investigation, Methodology, Visualization, Writing – original draft, Writing – review & editing. LP: Formal analysis, Methodology, Visualization, Writing – review & editing. JL: Supervision, Visualization, Writing – review & editing. SB: Funding acquisition, Supervision, Writing – review & editing. EM: Formal analysis, Visualization, Writing – review & editing. AM: Funding acquisition, Methodology, Supervision, Writing – review & editing. JW: Funding acquisition, Methodology, Supervision, Writing – review & editing. HM: Funding acquisition, Methodology, Supervision, Writing – review & editing.
