# Supplementary material for: Exploring veterinary students’ awareness and perception of zoonoses risks, infection control practices, and biosecurity measures in Ethiopia
Source: Front Vet Sci. 2024 Jul 9;11:1385849. doi: 10.3389/fvets.2024.1385849 (PMC11263103; doi:10.3389/fvets.2024.1385849)
Supplement: Supplementary file 1 [file Data_Sheet_1.PDF]

* 1. Please indicate your gender.

- Male
- Female

* 2. Please indicate your age (in years)

* 3. Which university are you studying at?

* 4. Year of study

- 3
- 4
- 5
- 6

* 5. Which region are you from?

- Addis Ababa (city)
- Afar Region
- Amhara Region
- Benishangul-Gumuz Region
- Dire Dawa (city)
- Gambela Region
- Harari Region
- Oromia Region
- Sidama Region
- Somali Region
- Southern Nations, Nationalities, and Peoples' Region
- Tigray Region

* 6. What level of importance do you attribute to the following statements?

	Extremely important	Very important	Moderately important	Slightly important	Not at all important
How important is collaboration between human and animal health-care providers for ensuring public health?					
How important is monitoring and detecting zoonoses outbreaks in animal populations for preventing human infection?					
How important is maintaining ecosystem and environmental health for protecting human and animal health?					
How important is preventing human encroachment for preventing emergence of new human and animal diseases?					
How important is addressing climate change for preventing emergence of new human and animal diseases?					

* 7. To what extent do you agree or disagree with the following statements?

	Strongly disagree	Disagree	Neither agree or disagree	Agree	Strongly agree
Consumers of animal products are at risk of contracting zoonoses					
Animals can serve as disease sentinels for human health					
Climate change directly and indirectly impacts human and animal health					
Veterinary and animal health professionals are at risk of contracting zoonoses					
Farmers and farm-workers are exposed to the risk of contracting zoonoses					

Biosecurity

* 8. What does the term 'biosecurity' mean to you?

- [ ] Preventing entry of pathogens or diseases onto farms
- [ ] Managing diseases / pathogens within farms
- [ ] Preventing exit of diseases / pathogens from farms
- [ ] General security to prevent theft of animals
- [ ] Protecting workers from disease
- [ ] Unsure
- [ ] Other (please specify)

* 9. How often do you change your overall, apron or overcoat?

- ○ I wear reusable clothing and change it every day
- ○ I wear reusable clothing and change it as soon as it is visually dirty
- ○ I wear reusable clothing and change it after specific ‘dirty’ work
- ○ I wear reusable clothing and change it after every cattle farm
- ○ I use a set of disposable clothing for each cattle farm
- ○ I use disposable clothing (i.e. different set per cattle farm)
- ○ I use clothing provided by the farmer

* 10. When performing surgeries, do you wear:

- ○ Disposable calving gowns
- ○ Washable calving gowns under ordinary circumstances, but disposable gown in case of known septic risk (e.g. emphysematous calf)
- ○ A washable calving gown

* 11. What do you use when washing your work clothes and linen? Please check all boxes corresponding to your choices.

- [ ] Detergent
- [ ] Disinfectant
- [ ] Soap
- [ ] Cold water
- [ ] Warm water
- [ ] Hot water

* 12. How do you take care of your work boots?

	After each farm	Before each farm	After and before each farm	Between two buildings on same farm	Only when they are visually dirty	Never
Brushing	○	○	○	○	○	○
Use of water jet	○	○	○	○	○	○
Cleaning with soap	○	○	○	○	○	○
Disinfection	○	○	○	○	○	○
Stepping through foot bath or stepping on foot mat	○	○	○	○	○	○

* 13. How often do you replace the following disposable materials and equipment?

	After each animal	After each herd / group	After each farm	Every day	Less frequently than every day
Needles for injections	○	○	○	○	○
Needles for sampling	○	○	○	○	○
Syringes	○	○	○	○	○
Scalpel and razor blades	○	○	○	○	○
Examination gloves	○	○	○	○	○
Full-arm veterinary gloves	○	○	○	○	○

* 14. How do you clean and take care of your reusable material and equipment (e.g. needles, syringes, scalpels, razors, gloves)?

- ◯ Cleaning
- ◯ Cleaning and soaking in disinfectant
- ◯ Cleaning and autoclaving (hot sterilization)
- ◯ Other (please specify)

* 15. How often do you wash your hands while working with animals?

- ◯ After each animal
- ◯ After each lot
- ◯ After each cattle farm
- ◯ Other (please specify)

* 16. How do you wash or clean your hands while working with animals?

- ☐ With clear water
- ☐ With a soap
- ☐ With an antibacterial soap
- ☐ With hand sanitizer
- ☐ Other (please specify)

* 17. How do you dry your hands?

- ◯ With re-usable towel
- ◯ With a paper towel
- ◯ Other (please specify)

* 18. Do you carry a yellow container for medical waste when you visit farms (i.e. in your car or work bag)?

- ◯ Yes
- ◯ No

* 19. How do you dispose of empty medicine packaging and vaccine flasks / vials?

- [ ] Yellow container for medical waste in the lab
- [ ] Domestic trash can
- [ ] Collected by a specialized company
- [ ] Glass waste container
- [ ] Government waste place
- [ ] Other (please specify)

* 20. Have you been vaccinated for zoonotic diseases?

- [ ] No, i have not been vaccinated
- [ ] Yes, for Tuberculosis
- [ ] Yes, for Tetanus
- [ ] Yes, for Rabies
- [ ] Other (please specify)

* 21. In each of the following scenarios, please indicate the biosecurity measures you would implement in examining animals and performing procedures.

	Hand wash after contact	Gloves only	Boot / shoes disinfection	Overalls only	Gloves and overalls	Gloves, overalls, respiratory mask and goggles	Not sure
Post mortem examination of a cow	○	○	○	○	○	○	○
Routine examination of a cow	○	○	○	○	○	○	○
Cow with acute watery diarrhoea	○	○	○	○	○	○	○
Aborted foetal material from a cow	○	○	○	○	○	○	○
Cow with dystocia (calving problems)	○	○	○	○	○	○	○
An animal with a multi-drug resistant urinary tract infection	○	○	○	○	○	○	○
Entering a farm	○	○	○	○	○	○	○

'One-Health'

22. What is your understanding of the term 'One-Health'?

- ◯ A collaborative approach of multiple health science professions to designing and implementing programmes, policies, legislation and research working together to achieve better public health outcomes.
- ◯ A collaborative framework involving the health of humans, animals, and ecosystems, including also environmental sustainability and socioeconomic stability
- ◯ A collaborative approach aiming to is the achievement of the highest attainable standard of health, well-being, and equity worldwide through judicious attention to the human systems—political, economic, and social—that shape the future of humanity and the Earth’s natural systems that define the safe environmental limits within which humanity can flourish.

* 23. How interested are you in the following?

	Very interested	Somewhat interested	Minimally interested	No Interest
'One Health' educational experiences	◯	◯	◯	◯
Lectures and courses taught by physicians	◯	◯	◯	◯
Lectures and courses taught by ecologists	◯	◯	◯	◯
Lectures and courses taught by biologists	◯	◯	◯	◯
Lectures and courses taught by interdisciplinary team of health professionals	◯	◯	◯	◯
Student exchange programs with human health medical colleges	◯	◯	◯	◯
Student exchange programs with public health schools	◯	◯	◯	◯
Research opportunities with non-veterinary 'One Health' practitioners	◯	◯	◯	◯
Networking opportunities with non-veterinary 'One Health' practitioners	◯	◯	◯	◯
Interdisciplinary professional conferences for veterinarians, physicians, and ecosystem health experts	◯	◯	◯	◯
Health internships hosted by faculties other than those within Colleges of Veterinary Medicine and Biomedical Sciences	◯	◯	◯	◯
Access to 'One Health' mentors who are non-veterinarians	◯	◯	◯	◯

* 24. Promotion of 'One Health' approach in Ethiopia.

	Yes	No	Not sure
Is the 'One-Health' approach adopted in your field?	◯	◯	◯
Do you think veterinary medicine has a role to play in preventing human diseases?	◯	◯	◯
'One-Health' implies better cooperation between doctors and veterinarians	◯	◯	◯
'One-Health' means working at the interaction between human and animal health and the ecosystem	◯	◯	◯
'One-Health' is a new approach to global health governance	◯	◯	◯
Is 'One Health' an important approach for you?	◯	◯	◯
Have you applied the 'One Health' approach in your own work/projects?	◯	◯	◯
Should Ethiopia organize more events and communicate more to spread and implement the 'One Health' approach?	◯	◯	◯
Should Ethiopia put in place 'One Health' national strategies and coordination structures?	◯	◯	◯
Should Ethiopia strengthen cooperation with international organization (WHO / FAO / OIE)?	◯	◯	◯
Should Ethiopia focus on specific diseases that develop at the interface between human / animal / environmental spheres?	◯	◯	◯

* 25. Please describe your knowledge and skills / approach to communicating and providing advice about zoonoses and human health.

	Yes	No
I am being trained to work with human health workers	○	○
I am being trained to communicate with human health workers	○	○
I would be able to regonise if a farmer is affected by a zoonosis	○	○
I would advise farmers to seek medical health when I realize their animal have an important zoonoses	○	○
I would be concerned about the health of farmers when treating their animals	○	○
I would be concerned about my health when treating sick animals	○	○

* 26. How do you feel about the following 'One Health' statements?

	Agree	Neutral	Disagree
'One Health' is an important approach that will shape the veterinary / animal health profession	○	○	○
As a veterinarian, it is my duty to promote the 'One Health' approach	○	○	○
There are enough practical frameworks for veterinarians to follow or promote issues about 'One Health'	○	○	○
It is important that my contribution to control / treat zoonoses will bring about a good outcome for the animal	○	○	○
It is important that my contribution to control / treat zoonoses will bring about a good outcome for farmer	○	○	○
I have a good understanding of protecting myself and my staff from potential zoonoses risks	○	○	○
I have a good understanding of anti-microbial stewardship guidelines for veterinarians	○	○	○

* 27. What is your opinion regarding animal–human health inter-professional collaboration?

	Strongly agree	Neutral	Disagree
Veterinarians are better equipped and more knowledgeable than physicians in understanding and approaching zoonotic cases	○	○	○
Having a human health referral system to consult veterinarians' animal-related knowledge would bring about positive changes to human health	○	○	○
I would be willing to collaborate with physicians to manage zoonotic cases that affect both my animal patients and human clients	○	○	○
I expect to be knowledgeable enough, in the future, to provide advice to human clients about preventing zoonotic diseases commonly transmitted from livetsock and companion animals	○	○	○
It will be an important part of my future work to provide advice to clients about preventing zoonotic diseases commonly transmitted from livestock and companion animals	○	○	○
A veterinarian should always ask clients if there are any immune-compromised and immune-deficient members, pregnant, young or elderly members living with the livetsock as part of the basic information	○	○	○

* 28. To what extent is antibiotic resistance a problem in Ethiopia?

- ◯ Not at all a problem
- ◯ Minor problem
- ◯ Moderate problem
- ◯ Serious problem

* 29. If it is a problem, what do you think is influencing antibiotic resistance in Ethiopia?

- ☐ Antibiotic prescribing behaviour
- ☐ Misuse of antibiotics
- ☐ Non-observing of withdrawal periods
- ☐ Use of antibiotics in feeds
- ☐ Symptomatic treatment of animals (without lab results and guidance)
- ☐ Sub-standard drugs
- ☐ Over-use of drugs
- ☐ Other (please specify)

* 30. To what extent do you think addressing antibiotic resistance is currently a priority in Ethiopia?

- ◯ Not a priority
- ◯ Low priority
- ◯ Medium priority
- ◯ High priority
- ◯ Essential

* 31. What are your personal beliefs about antibiotic / antimicrobial use and resistance?

	Agree	Neutral	Disagree
Antimicrobials are overused nationally	◯	◯	◯
Better use of antimicrobials will reduce problems with antimicrobial resistant organisms	◯	◯	◯
Knowledge of appropriate use of antibiotics / antimicrobials is important in my veterinary career	◯	◯	◯
I would like more education on antibacterial / antimicrobial resistance	◯	◯	◯
New antimicrobials will be developed in the future that will keep up with the problem of resistance	◯	◯	◯
Poor infection control practices by veterinary professionals causes spread of antimicrobial resistance	◯	◯	◯
Inappropriate use of antimicrobials causes antimicrobial resistance	◯	◯	◯
Antimicrobial resistance will affect animal health and production	◯	◯	◯
I have sufficient knowledge on antibiotics use for future clinical practice	◯	◯	◯
Antibiotics used to treat animals can remain within their tissues	◯	◯	◯

* 32. How important do you think would the following sources of information be, in your future work, in determining your antimicrobial choices?

	Not at all important	Slightly important	Moderately important	Very important	Extremely important
Pharmaceutical company representatives	◯	◯	◯	◯	◯
Label or package inserts	◯	◯	◯	◯	◯
Peer-reviewed scientific literature	◯	◯	◯	◯	◯
Peers within my practice / office	◯	◯	◯	◯	◯
Peers beyond my practice / office	◯	◯	◯	◯	◯
Clinicians and pharmacists	◯	◯	◯	◯	◯
Veterinary Information Network (VIN)	◯	◯	◯	◯	◯
Online resource (e.g. blogs, media posts or web searches)	◯	◯	◯	◯	◯
Textbooks or drug handbooks	◯	◯	◯	◯	◯
Applications on a smart phone or tablet	◯	◯	◯	◯	◯
Online formulary (list of medicines)	◯	◯	◯	◯	◯

* 33. How confident do you feel that your veterinary medicine studies have prepared you to do the following upon graduation?

	Completely confident	Fairly confident	Somewhat confident	Slightly confident	Not at all confident
To know when to start antimicrobial therapy	◯	◯	◯	◯	◯
How to select the best antimicrobial for a specific infection	◯	◯	◯	◯	◯
How to select the correct dosing	◯	◯	◯	◯	◯
How to select the right duration of treatment for specific infections	◯	◯	◯	◯	◯
To describe the correct spectrum of antimicrobial therapy for different antimicrobials (what is covered by each drug)	◯	◯	◯	◯	◯
Understand the basic mechanisms of antimicrobial resistance	◯	◯	◯	◯	◯
How to streamline or deescalate antimicrobial therapy	◯	◯	◯	◯	◯
How to interpret antibiograms	◯	◯	◯	◯	◯
How to find reliable sources of information to treat infections	◯	◯	◯	◯	◯
How to handle a clients who demands antimicrobial therapy that is not indicated	◯	◯	◯	◯	◯

Sources of information

* 34. What are your main sources of information about...

	Education curriculum	Collegues and friends	Mass media (TV, Radion newspaper)	Scientific journals	Online searches (i.e Google)	Social networks (i.e. Facebook, Twitter, Instgram, LinkedIn)	Government communication	Professional association and subscriptions
Zoonoses	☐	☐	☐	☐	☐	☐	☐	☐
Biosecurity measures	☐	☐	☐	☐	☐	☐	☐	☐
One-Health	☐	☐	☐	☐	☐	☐	☐	☐
Antibiotic resistance	☐	☐	☐	☐	☐	☐	☐	☐
New treatments	☐	☐	☐	☐	☐	☐	☐	☐
Animal diseases outbreaks	☐	☐	☐	☐	☐	☐	☐	☐

* 35. How easy is it to get information on the following topics?

	Very easy	Easy	Moderately easy	Difficult
Zoonoses	○	○	○	○
Personal biosecurity measures	○	○	○	○
Farm biosecurity measures	○	○	○	○
One-Health	○	○	○	○
Antibiotic resistance	○	○	○	○
New treatments	○	○	○	○
Animal disease outbreaks	○	○	○	○

* 36. How easy is it to get information from the above sources?

	Very easy	Easy	Moderately Easy	Difficult
Training curriculum	◯	◯	◯	◯
Collegues and friends	◯	◯	◯	◯
Mass media (TV, Radion newspaper)	◯	◯	◯	◯
Scientific journals	◯	◯	◯	◯
Online searches i.e google	◯	◯	◯	◯
Social networks (Facebook, twitter, instgram, LinkdIn)	◯	◯	◯	◯
Government communication	◯	◯	◯	◯
Professional association and subscriptions	◯	◯	◯	◯

* 37. In your opinion, what are the **5 most important personal attributes (characteristics)** in a veterinary surgeon?

	First attribute	Second attribute	Third attribute	Fourth attribute	Fifth attribute
Confidence	○	○	○	○	○
Friendliness	○	○	○	○	○
Knowledge about veterinary medicine and surgery	○	○	○	○	○
Cleanliness	○	○	○	○	○
Good at explaining technical terms	○	○	○	○	○
Professional appearance	○	○	○	○	○
Compassion for patients	○	○	○	○	○
Compassion for owners	○	○	○	○	○
Good communication skills	○	○	○	○	○
A likeable personality	○	○	○	○	○
Patience	○	○	○	○	○
Good listening skills	○	○	○	○	○
Recognises own limitations and knows when to seek advice	○	○	○	○	○
Clear about cost of treatment	○	○	○	○	○
Ability to work in a team	○	○	○	○	○
Honesty	○	○	○	○	○
Politeness	○	○	○	○	○
Decisiveness	○	○	○	○	○
Good with animals	○	○	○	○	○
Good practical skills	○	○	○	○	○

* 38. In your opinion, what are the **5 least important personal attributes (characteristics)** in a veterinary surgeon?

	First attribute	Second attribute	Third attribute	Fourth attribute	Fifth attribute
Confidence	○	○	○	○	○
Friendliness	○	○	○	○	○
Knowledge about veterinary medicine and surgery	○	○	○	○	○
Cleanliness	○	○	○	○	○
Good at explaining technical terms	○	○	○	○	○
Professional appearance	○	○	○	○	○
Compassion for patients	○	○	○	○	○
Compassion for owners	○	○	○	○	○
Good communication skills	○	○	○	○	○
A likeable personality	○	○	○	○	○
Patience	○	○	○	○	○
Good listening skills	○	○	○	○	○
Recognises own limitations and knows when to seek advice	○	○	○	○	○
Clear about cost of treatment	○	○	○	○	○
Ability to work in a team	○	○	○	○	○
Honesty	○	○	○	○	○
Politeness	○	○	○	○	○
Decisiveness	○	○	○	○	○
Good with animals	○	○	○	○	○
Good practical skills	○	○	○	○	○
